# Coagulase-negative staphylococci from bovine milk: Antibiogram profiles and virulent gene detection

**DOI:** 10.1186/s12866-024-03415-0

**Published:** 2024-07-18

**Authors:** Yared Abate Getahun, Solomon Lulie Abey, Achenef Melaku Beyene, Mequanint Addisu Belete, Tesfaye Sisay Tessema

**Affiliations:** 1 Livestock and Fishery Research Center, Arba Minch University, P.O.BOX: 21, Arba Minch, Ethiopia; 2 Department of Pathobiology, College of Veterinary Medicine and Animal Science, University of Gondar, Gondar, Ethiopia; 3 Department of Veterinary Pharmacy, College of Veterinary Medicine and Animal Sciences, University of Gondar, Gondar, Ethiopia; 4 Department of Veterinary Laboratory Technology, College of Agriculture and Natural Resource, Debre Markos University, Debre Markos, Ethiopia; 5 Institute of Biotechnology, Addis Ababa University, Addis Ababa, Ethiopia

**Keywords:** Antibiogram profile, Bovine milk, Coagulase-negative *Staphylococcus* species, Virulent gene

## Abstract

**Background:**

Coagulase-negative *Staphylococcus* species are an emerging cause of intramammary infection, posing a significant economic and public health threat. The aim of this study was to assess the occurrence of coagulase-negative *Staphylococcus* species in bovine milk and dairy farms in Northwestern Ethiopia and to provide information about their antibiotic susceptibility and virulence gene profiles.

**Methods:**

The cross-sectional study was conducted from February to August 2022. Coagulase-negative *Staphylococcus* species were isolated from 290 milk samples. Species isolation and identification were performed by plate culturing and biochemical tests and the antimicrobial susceptibility pattern of each isolate was determined by the Kirby-Bauer disc diffusion test. The single-plex PCR was used to detect the presence of virulent genes. The STATA software version 16 was used for data analysis. The prevalence, proportion of antimicrobial resistance and the number of virulent genes detected from coagulase-negative *Staphylococcus* species were analyzed using descriptive statistics.

**Results:**

Coagulase-negative *Staphylococcus* species were isolated in 28.6%, (95% CI: 23.5–34.2) of the samples. Of these, the *S. epidermidis*, *S. sciuri*, *S. warneri*, *S. haemolyticus*, *S. simulans*, *S. chromogens*, *S. cohnii*, and *S. captis* species were isolated at the rates of 11, 5.2, 3.4, 3.1, 3.1, 1, 1, and 0.7% respectively. All the isolates showed a high percentage (100%) of resistance to Amoxicillin, Ampicillin, and Cefotetan and 37.5% of resistance to Oxacillin. The majority (54.2%) of coagulase-negative isolates also showed multidrug resistance. Coagulase-negative S*taphylococcus* species carried the *icaD*, *pvl*, *mecA*, *hlb*, *sec*, and *hla* virulent genes at the rates of 26.5%, 22.1%, 21.7%, 9.6%, 9.6% and 8.4% respectively.

**Conclusion:**

The present study revealed that the majority of the isolates (54.2%) were found multidrug-resistant and carriage of one or more virulent and enterotoxin genes responsible for intramammary and food poisoning infections. Thus, urgent disease control and prevention measures are warranted to reduce the deleterious impact of coagulase-negative species. To the best of our knowledge, this is the first study in Ethiopia to detect coagulase-negative *Staphylococcus* species with their associated virulent and food poisoning genes from bovine milk.

**Supplementary Information:**

The online version contains supplementary material available at 10.1186/s12866-024-03415-0.

## Background

*Staphylococcus* species are the major pathogen of lactating dairy cows causing a significant reduction in milk yield and posing risk to the public health [27, 45]. It can be classified as coagulase-positive and coagulase-negative *Staphylococcus* species based on their ability to clot the rabbit plasma, a critical diagnostic step in clinical microbiology [44].

Coagulase-negative *Staphylococcus* (CoNS) species were previously considered less pathogenic because they were only reported in sub-clinical mastitis cases and thus received less attention. However, a growing body of evidence suggests that CoNS species have been isolated from clinical mastitis infections, and they are regarded as emerging pathogens of bovine intramammary infections [13, 46, 59]. The most common CoNS species reported from intramammary infections are *S. chromogens*, *S. epidermidis*, *S. haemolyticus*, *S. sciuri*, *S. simulans*, *S. cohnii*, *S. xylosus*, *S. warneri*, *S. captis* and *S. equorum* [8, 35, 47].

Several virulence factors, such as the enterotoxin (SE) genes (*sea* to *seQ*), the toxic shock syndrome toxin-1 gene (*tsst-1*), clumping factor (*clfA* to *clfD*), intracellular adhesion gene (*icaA* and *icaD*), hemolysin toxin gene (*hla*, *hlb*, *hld*, *Y-hlg*) the panton valentine leukocidin (*pvl*) gene and genes encoding drug resistance (*mecA and mecC*) were originally identified and characterized in *Staphylococcus aureus*; but now these virulent genes are detected in CoNS species. These virulent factors are responsible for the colonization and pathogenesis of the mammary gland as well as food poisoning [24, 25, 30, 38].

A lot of reports were available on *S. aureus* as a cause of mastitis and food intoxication; however, information on virulence factors of CoNS species for intramammary infection and food poisoning was scarce globally [37]. Furthermore, the level of occurrence and antibiotic resistance patterns of these bacterial species are not well studied in Ethiopia. In fact, no published reports are available on the virulent genes of CoNS species from bovine milk. Therefore, the study aimed to determine the antimicrobial susceptibility and virulent gene profile of coagulase-negative *Staphylococcus* species from bovine milk samples.

## Materials and methods

### Study area

**Fig. 1:**
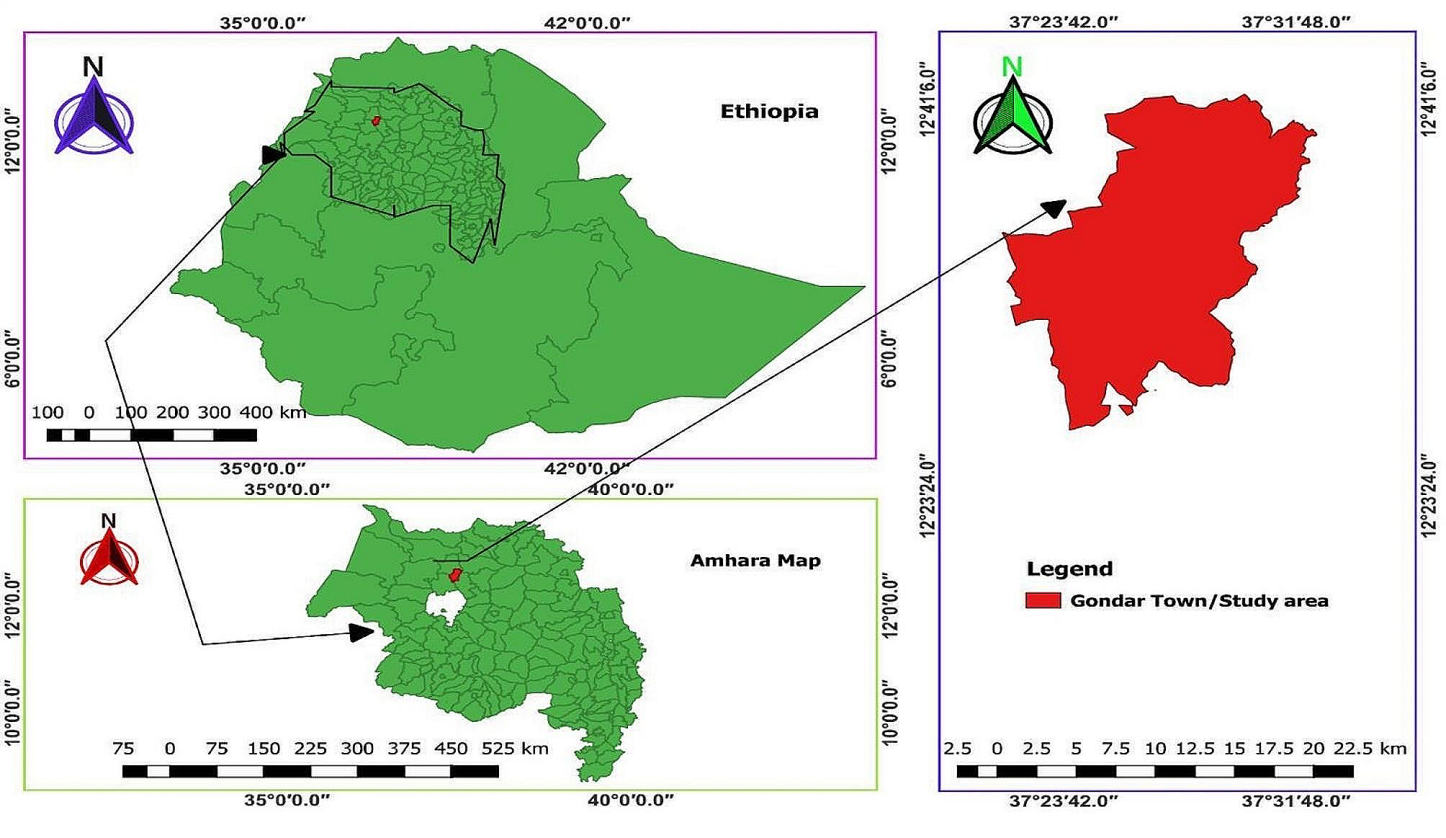
The geographical location of the study area

The study was carried out in Gondar City (Fig. 1); which is located 728 km northwest of Addis Ababa, at 12° North latitude, 37° 28` East longitude, with altitudes ranging from 1802 to 2200 m above sea level. According to the Gondar City livestock and fishery resource office, 2022 report during commencement of the study, the city consists of 42,929 cattle, 16,090 sheep, 7,614 goats, 6,461 donkeys, 516 horses, 35 mules, 13,468 bee colonies, and 126,061 poultries. The dairy cows are managed in both an intensive and semi-intensive systems [9].

### Study design, and sampling methods

A cross-sectional study was designed to collect milk samples from February to August 2022 from Zebu and cross-Holstein Friesian breed lactating cows. The sample size was determined based on Slovon’s formula [3].

n = N/(1 + N*e^2^)

Where, n = is the number of lactating cows selected for sample collection, N = the total number of lactating cows found in the study area, and e = the margin of error. In Gondar town, around 1060 lactating cows were found at the farm level and a 0.05 margin of error (95% CI) was taken for the study. Therefore, based on the above formula the milk samples were collected from 290 lactating cows. The dairy farms were chosen purposively based on the herd size; farms having three and more lactating cows were considered for sample collection and the milk samples were collected from all lactating cows in selected dairy farms.

The milk samples were collected following the standard procedures outlined in the National Mastitis Council [43]. The animals selected for milk sample collection were properly examined for the presence of cardinal signs of inflammation through visual inspection and palpation of the udder and visual inspection of milk for the presence of flakes, clots, and discoloration. A composite milk of about 10 ml was collected from all quarters of lactating cows using a universal bottle after discarding the first three milk streams and transported to the University of Gondar, Veterinary Microbiology Laboratory for bacteriological analysis.

### Isolation and identification protocol

The milk samples taken from lactating cows were cultured directly on mannitol salt agar (MSA) (Alpha Chemica, India) and incubated aerobically at 37 °C for 24–48 h. The cultures that produced pink and white colonies on mannitol salt agar were considered either *Staphylococcus* species or *Micrococcus* species. Thus, the oxidation fermentation test (OF) test was performed according to procedures outlined by Varghese and Joy [58] to differentiate the *Staphylococcus* species from the *Micrococcus* species.

Those Staphylococcal colonies that grew on the mannitol salt agar; were positive for catalase tests, fermentative on the OF media, had characteristic cluster cocci shape on Gram stain were further differentiated into coagulase-positive and negative *Staphylococcus* species based on their ability to clot the rabbit plasma. Thus, the tube coagulase test was performed by mixing 4–5 pure staphylococcal colonies grown on tryptone soya agar (HiMedia, Laboratories, Ltd., India) with 0.5 mL of the rabbit plasma (National Veterinary Institute, Debre Zeit, Ethiopia) according to Fernandes Queiroga Moraes et al. [22].

A Pure colony of the CoNS species was sub-cultured on blood agar (HiMedia Laboratories Pvt. Ltd., India) containing 7% sheep blood to observe the hemolysis pattern and colony morphology. The identification of CoNS was performed based on the colony characteristics on mannitol salt agar, hemolysis characteristics on blood agar, the ability to utilize urea and maltose and sucrose sugar fermentation tests. The oxidase test was also performed by rubbing four to five pure colonies grown on nutrient agar with a cotton swab soaked with the oxidase reagent (Table 1).

**Table 1:** Biochemical and plate culturing methods for CoNS identification

CoNS species	CoNS species identification tests						Reference
	MSA	BA	Urease test	MSFt	SSFt	Oxidase test	
*S. epidermidis*	-	-/+	+	+	+	-	[15]
*S. haemolyticus*	+	+	-	+	+	-	[50]
*S. simulans*	+	+/-	+	-/+	+	-	[15].
*S. captis*	+	-	-	-	+/-	-	[31]
*S. cohnii*	+	-	-/+	+	-	-	[50]
*S. warneri*	+	-	+	+	+	-	[31]
*S. sciuri*	-	-	-	-/+	+	+	[55]
*S. chromogenes*	-	-	+	-	+	-	[18, 39]

*Key:* CoNS- coagulase-negative *Staphylococcus* species; MSA- Mannitol salt agar; BA- blood agar; MSFt- maltose sugar fermentation test; SSFt- sucrose sugar fermentation test; + = positive; - = negative for the test; +/-= majority are positive but some are negative; - /+= majority of the test organisms or strains are negative for the test but some species or strains show a positive result

Phenotypically isolated and purified CoNS species isolates were preserved in tryptone soya (HiMedia Laboratories Pvt. Ltd, India) broth mixed with 25% glycerol at -20◦C until molecular analysis started.

### Antimicrobial susceptibility pattern of coagulase-negative *Staphylococcus* species

The antimicrobial susceptibility pattern of CoNS species isolates was examined using the Kirby Bauer disc diffusion methods, following the guidelines of the clinical laboratory standard institute [12]. The standardized bacterial inoculum was prepared by dissolving the CoNS species colony in 0.85% normal saline until the turbidity of the suspension was equivalent to 0.5 Mac Farland standards. A sterile cotton swab was soaked to the bacterial suspension and streaked onto the Mueller–Hinton Agar (MHA) plate (HiMedia Laboratories, Pvt. Ltd., India). The plates were left dry for 5 min before disc placement.

The antimicrobial discs (all from Oxoid, UK), with the following disc concentration; Gentamicin (GEN,10 µg), Sulphamethoxazole-trimethoprim (SXT, 2 µg), Erythromycin (E, 10 µg), Ampicillin (AMP, 10 µg), Amoxicillin (AMX, 10 µg), Penicillin G (P10, 1U), Cefotetan (CTT, 30 µg), Oxacillin (OXC, 1 µg), Tetracycline (TE, 30 µg) were used for antimicrobial susceptibility tests. The diameter of the zone of inhibition produced by each antimicrobial disc was compared with the clinical laboratory standard institute [12] standards to determine whether the CoNS species isolates were susceptible or resistant to particular antimicrobial discs. Coagulase-negative *Staphylococcus* species isolates were considered multidrug resistant (MDRS) when the isolates showed resistance to three or more antimicrobial classes [16]. Phenotypic methicillin resistance of CoNS species isolates was checked using Oxacillin discs.

### Virulent gene detection

#### Bacterial DNA extraction

A pure colony of CoNS species isolates were cultured for 24 h in brain heart infusion broth (BHI) (HiMedia Laboratories Pvt. Ltd, India). One ml of the broth was transferred to 1.5 ml of the Eppendorf tube and spun at 10,000 rpm for 10 min. After discarding the supernatant, the bacterial pellets were washed with 1 ml of phosphate buffer saline (PBS) by spinning at 10,000 rpm for 10 min and discarding the supernatant completely. The bacterial DNA extraction was performed using the EZ-10 spin column genomic DNA minipreps kit (Bio Basic Inc, Canada) according to the manufacturer’s instructions. The quality of extracted DNA was checked with NanoDrop 2000 spectrophotometer.

### Polymerase chain reaction assay

Single-plex polymerase chain reaction assay was performed for the detection of 14 virulent genes including; the six enterotoxins, SE genes, *(sea*, *seb*, *sec*, *sed*, *see*, *seh)*, the toxic shock syndrome toxin-1 (*tsst-1*), intercellular adhesion gene D (*icaD*), hemolysin toxin (*hla and hlb*,* Y-hlg*), the panton valentine leukocidin (*pvl*) gene, as well as genes encoding drug-resistant (*mecA and mecC*) using gene-specific oligonucleotide primers (Table 2).

**Table 2:** Primers used for the amplification of virulent genes of CoNS species

Target Gene	Gene code	Primer Sequence (5`-3`)	Primer length(bp)	Amplicon Size (bp)	Reference
Enterotoxins	*sea*	F: ATTAACCGAAGGTTCTGTAGA	21	552 bp	[37]
		R: TTGCGTAAATCTGAATT	17		
	*seb*	F: TGTATGTATGGAGGTGTAAC	24	270 bp	
		R: ATAGTGACGAGTTAGGTA	18		
	*sec*	F: CTTGTATGTATGGAGGAATAACAA	24	284 bp	[20]
		R- TGCAGGCATCATATCATACCA	21		
	*sed*	F: CTAGTTTGGTAATATCTCCT	20	317 bp	[37]
		R: TAATGCTATATCTTATAGGG	20		
	*see*	F: TAGATAAAGTTAAAACAAGC	20	170 bp	
		R: TAACTTACCGTGGACCCTTC	20		
	*she*	F: CACATCATATGCGAAAGCAGA	21	617 bp	
		R: CCTTTTAAATCATAAATGTCGAATGA	26		
Hemolysins	*hlb*	F: GCCAAAGCCGAATCTAAGAAAG	22	495	[2]
		R: ATCATGTCCAGCACCACAA	19		
	*hla*	F: GGTTTAGCCTGGCCTTC	17	550	[42]
		R: CATCACGAACTCGTTCG	17		
Methicillin resistance gene	*mecA*	F: TCCAGATTACAACTTCACCAGG	22	162	[37]
		F: CCACTTCATATCTTGTAACG	20		
	*mecC*	R: GAAAAAAAGGCTTAGAACGCCTCC	24	138 bp	[37]
		R: GAAGATCTTTTCCGTTTTCAGC			
Toxic shock syndrome toxin-1	*tsst-1*	F: ACCCCTGTTCCCTTATCATC	20	326	[2, 14]
		R: TTTTCAGTATTTGTAACGCC	20		
Intercellular adhesion	*icaD*	F: AAACGTAAGAGAGGTGG	17	381	[5]
		R: GGCAATATGATCAAGATAC	19		
Gamma hemolysins	*Y-hlg*	R: TGTGGATCCGTCATTCATTG	20	937 bp	[37]
		F: CCAATCCGTTATTAGAAAATGC	22		
Cytotoxic genes	*pvl*	R: CCATAGACGTAGCAACGGAT	20	118 bp	[37]
		F: TTACACAGTTAAATATGAAGTGAACTGGA	29		

*Key: sea*, *seb*,* sec*, *sed*, *see*,* seh* are enterotoxin A, B, C, D, E and H genes respectively. *hla*-hemolysin A; *hlb*- hemolysin B; *tsst- 1*- toxic shock syndrome toxin − 1; *mecA* and *mecC*- methicillin resistance gene A and C; *pvl*- the panton valentine leukocidin and *Y-hlg*-gamma hemolysin gene

The PCR reaction mixture of 20 µl was prepared by adding a similar amount of 2 µl of 10x reaction buffer (Solis BioDyne, Estonia), 0.2 µl of dNTPs, 0.2 µl of FIREPol DNA polymerase (SolisBioDyne, Estonia) for all PCR reaction; but the amount of MgCl_2_, primers, bacterial genomic DNA and nuclease-free water added to each PCR reaction was vary (Table 3).

**Table 3:** The PCR reaction components used for amplification of virulent genes in coagulase-negative *Staphylococcus* species isolates

Virulent genes	Amount of the PCR reaction components in µL							Annealing ^o^T in ^o^C
	NFW	10x reaction buffer	dNTPs	MgCl_2_	Primer (Fw + Rv)	DNA-polymerase	Sample DNA	
*hla*	11.6	2	0.2	2	1	0.2	3	59
*hlb*	12.1	2	0.2	1.5	1	0.2	3	55
*icaD*	12.1	2	0.2	1.5	1	0.2	3	48
*pvl*	12.1	2	0.2	1.5	1	0.2	3	55
*sec*	11.9	2	0.2	1.5	1.2	0.2	3	53
*tsst-1*	11.6	2	0.2	2	1	0.2	3	55
*mecA*	12.1	2	0.2	1.5	1	0.2	3	51

*Key:* NFW-nuclease free water; dNTP- deoxynucleotide triphosphate; *hla*-hemolysin A gene; *hlb*- hemolysin B gene; *icaD*-intracellular adhesion gene D; *pvl*- the panton-valentine leukocidin gene; *mecA*- gene coding methicillin resistance; *tsst-1*- toxic shock syndrome toxin − 1 gene; *sec*-enterotoxin C gene

Prima 96 thermocycler (HiMedia, Laboratories Pvt. Ltd., India) was used for the amplification of virulent genes in CoNS species isolates with an initial denaturation at 95 ^o^C for 5 min, denaturation at 95 ^o^C for 30 s, extension at 72 ^o^C for 1 min and final extension at 72 ^o^C for 8 min. All virulent gene amplification was performed for 35 PCR cycles except *pvl* gene amplification which was performed for 37 PCR cycles.

The electrophoresis of each PCR amplified product was performed using 1.5% agarose gel (HiMedia Laboratories Pvt. Ltd., India) and stained with ethidium bromide. A molecular ladder of 100 bp was used to compare the size of the amplicon product. The gel product was examined by the gel documentation system (UVITEC, Cambridge, UK) under ultraviolet (UV) illumination.

### Data management and analysis

The data was recorded in a Microsoft Excel spreadsheet. The STATA version 16 software was used to analyze the data. Descriptive statistics were used to analyze the isolation rate, proportion of antimicrobial susceptibility and virulent gene profile of the CoNS species isolates.

## Results

### Species isolation and identification

The milk samples were collected from 290 lactating cows. The overall isolation rate of CoNS species in the study area was 28.62%, 83/290 (95% CI: 23.5–34.2). The isolation rate of each CoNS species from the milk sample described in (Table 4).

**Table 4:** The prevalence of coagulase-negative *Staphylococcus* species isolated from 290 milk samples

CoNS species	No. positives, *N* = 290	Prevalence (%, CI)
*S. epidermidis*	32	11%, 7.7–15.2
* S. sciuri*	15	5.2%, 2.9–8.4
* S. warneri*	10	3.4%, 1.7–6.2
* S. haemolyticus*	9	3.1%, 1.4–5.8
* S. simulans*	9	3.1%, 1.4–5.8
* S. chromogens*	3	1%, 0.2–2.99
* S. cohnii*	3	1%, 0.2–2.99
* S. captis*	2	0.7%, 0.08–2.4

*Key:* N = total number of samples, CI- confidence interval

### Antibiogram profiles of the coagulase-negative *Staphylococcus *species

The phenotypic methicillin resistance capacity of each CoNS species was tested using Oxacillin antibiotic discs; thus, 37.3% of CoNS species showed phenotypic methicillin resistance to Oxacillin whereas 21.7% of CoNS isolates showed genotypic methicillin resistance.

The CoNS species isolates showed 51.8, 27.7, 49.4, 37.3, 43.4, and 39.8% resistance to Penicillin G, Gentamycin, Erythromycin, Oxacillin, sulphamethoxazole-trimethoprim, and Tetracycline discs respectively. The CoNS species isolates also showed 100% resistance to Ampicillin, Amoxicillin, and Cefotetan antimicrobials discs (Table 5).

**Table 5:** The proportion of CoNS isolates exhibiting resistance to different antimicrobial discs

Antimicrobial discs	The proportion of CoNS species showing antimicrobial resistance (%)								
	*S. epidermidis*, *N* = 32	*S. haemolyticus*, *N* = 9	*S. sciuri*, *N* = 15	*S. simulans*, *N* = 9	*S. warneri*, *N* = 10	*S. chromogens*, *N* = 3	*S. cohnii*, *N* = 3	*S. captis*,*N = 2*	Overall resistance, *N* = 131
Penicillin G (P, 10 units)	60	55.5	46.7	44.4	30	66.7	66.7	50	51.8
Gentamycin (GEN, 30 µg)	21.9	33.3	53.3	0	40	0	33.3	0	27.7
Amoxicillin (AMX, 10 µg)	100	100	100	100	100	100	100	100	100
Erythromycin (E, 30 µg)	40.6	33.3	26.7	100	100	33.3	33.3	50	49.4
Oxacillin (OXC, 1 µg)	34.4	33.3	26.7	55.5	30	33.3	100	50	37.3
Sulphamethoxazole-trimethoprim (SXT, 30 µg)	25	22.2	20	100	70	100	66.7	100	43.4
Tetracycline (TE, 30 µg)	25	55.5	53.3	44.4	30	66.7	100	0	39.8
Ampicillin (AMP, 10 µg)	92	100	100	100	100	100	100	100	100
Cefotetan (CTT, 30 µg)	100	100	100	100	100	100	100	100	100

**Fig. 2:**
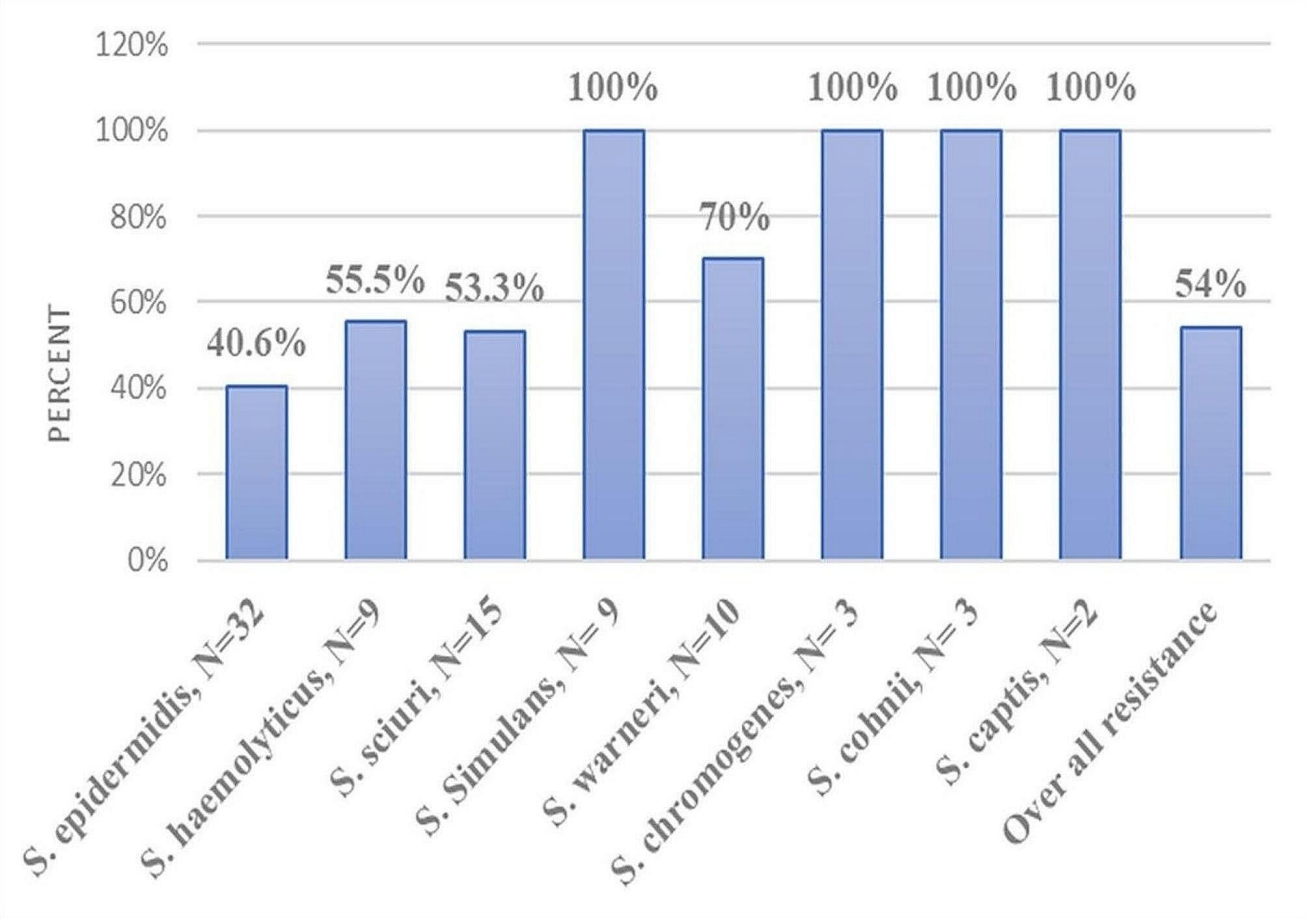
The proportion of *Staphylococcus* species isolates showed multidrug resistance to three or more antimicrobial classes

The present study also revealed, 54% of CoNS species isolates showed multidrug resistance. All isolates of the *S. simulans*, *S. chromogens*, *S. cohnii*, *S. captis* species showed multidrug resistance towards three or more antimicrobial classes (Fig. 2).

### Virulent Genes of coagulase-negative *Staphylococcus *species

**Fig. 3:**
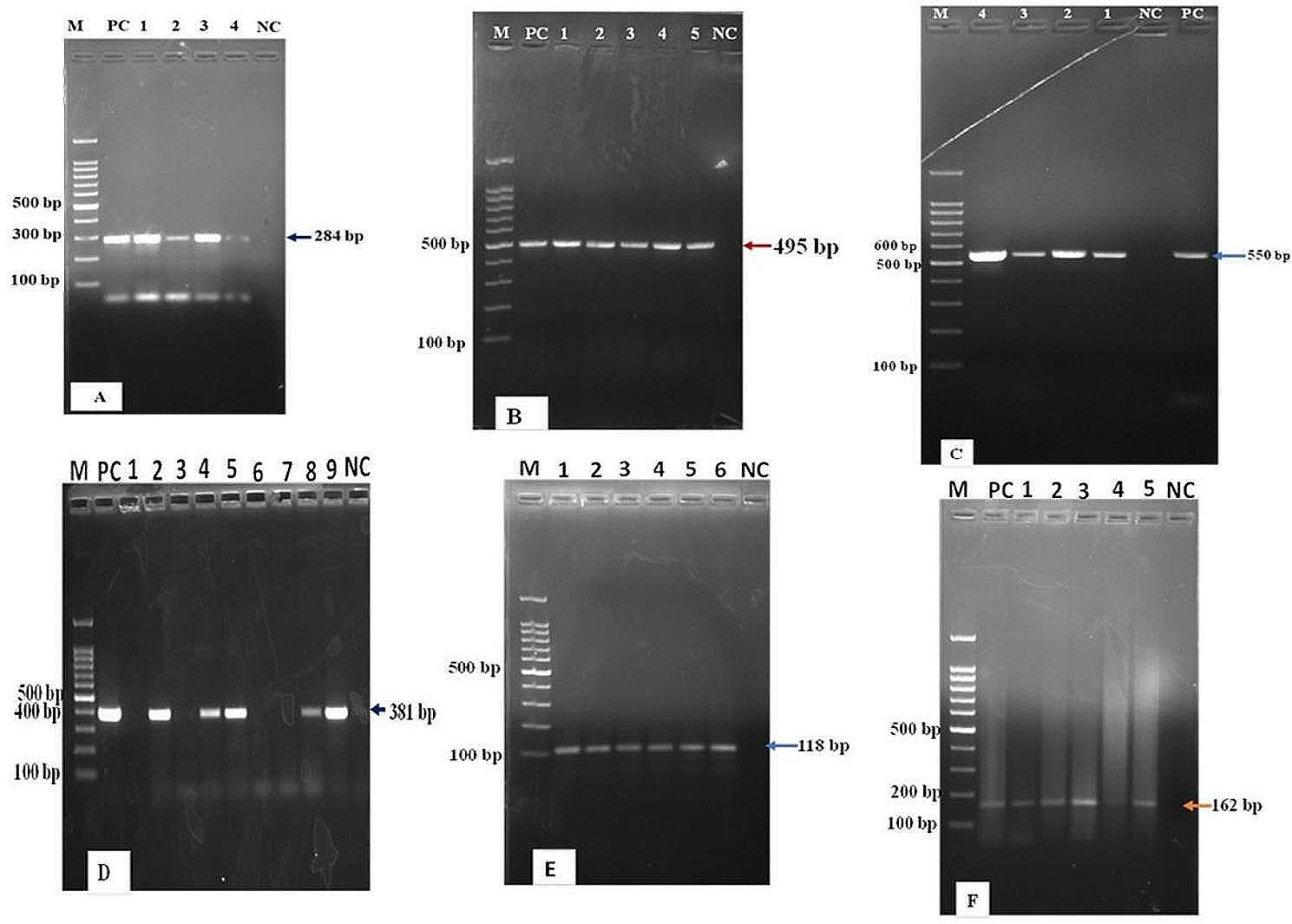
Lane M: Molecular marker; PC: positive control; NC: negative control. A and C, lanes 1 to 4 are positive tests for *sec* and *hla* genes respectively. B and F, lanes 1–5 indicate positive tests for *hlb* and *mecA* genes respectively. E, Lanes 1–6 are positive test results for *pvl* gene. D, lanes 2, 4,5,8,9 are positive and lanes 1,3,6,7 are negative for the *icaD* gene

Among the 14 virulent genes tested in CoNS species isolates, six virulent genes; namely the *icaD*, *pvl*, *mecA*, *hlb*, *sec* and *hla* genes were detected at the rates of 26.5, 22.1, 21.7, 9.6, 9.6 and 8.4% respectively from 83 CoNS species isolates (Fig. 3).

All CoNS species isolates except the *S. captis* were found to harbor one or more virulent genes. Genes coding for food poisoning (*sec*) were detected from *S. haemolyticus*, *S. sciuri* and *S. warneri* species isolates. The *icaD* genes were the most frequently detected followed by *mecA* and the *pvl* genes. The present study also revealed, except for *S. captis* the intracellular adhesion (*icaD*) genes were detected in all CoNS species isolates. The *S. epidermidis* isolates were carried only the *icaD* and *mecA* genes; whereas the *S. simulans* and *S. cohnii* species were found harboring five virulent genes (Table 6).

**Table 6:** The proportion of virulent genes detected from CoNS species isolates

CoNS species isolates	The proportion of virulent genes detected from CoNS species (%)					
	*hla*	*hlb*	*sec*	*icaD*	*pvl*	*mecA*
*S. epidermidis*, *N* = 32	-	-	-	21.6	-	21.9
* S. haemolyticus*, *N* = 9	33.3	33.3	22.2	22.2	-	-
*S. sciuri*, *N* = 15	-	-	26.7	26.7	20	-
*S. simulans*, *N* = 9	33.3	44.4	-	33.3	44.4	22.2
*S. warneri*, *N* = 10	-	-	20	30	55.5	44.4
*S. chromogens*, *N* = 3	-	-	-	33.3	66.7	33.3
*S. cohnii*, *N* = 3	33.3	33.3	-	66.7	33.3	33.3
Total CoNS	8.4	9.6	9.6	26.5	18.1	21.7

*Key* CoNS- coagulase-negative *Staphylococcus* species; *hla*- hemolysin A gene; *hlb*- hemolysin B gene; *sec*- enterotoxin C; *icaD*- intracellular adhesion D; *pvl*- panton valentine leukocidin gene; *mecA*- gene coding methicillin resistance; N- number of coagulase-negative *Staphylococcus* species isolates

## Discussion

Coagulase-negative staphylococcal infection is an emerging disease of lactating dairy cows and causes a significant reduction in milk yield and threatens public health [17, 30]. Virulent gene detection from coagulase-negative *Staphylococcus* species is the first report in Ethiopia.

The present study revealed that CoNS species were isolated at a rate of 28.6%, (83/290), 95% CI: 23.5–34.2). The result was lower than the report of Enquebaher et al. [19] in the Tigray region, Zeryehun and Abera [61] in selected districts of Eastern Harrarghe, Balemi et al. [6] in two pastoral districts Southern Ethiopia who reported 51.6%, 34.2% and 39% prevalence respectively, and higher than the reports of Gizaw et al. [28], Abunna et al. [1], Fesseha et al. [23] who reported 9.6%, 15% and 12.5% prevalence of CoNS species in Oromia, in and around Addis Ababa and in Modjo Town and Suburbs, Central Oromia respectively. A higher isolation rate of CoNS species from milk might be associated with the CoNS being prominent biofilm producers [29, 36] which enhances the bacteria to resist antibiotics and evade immune system mounting. In addition to this, CoNS are mainly recovered from subclinical mastitis, which helps the pathogen remain undetected and not treated with antimicrobials.

The current study revealed all CoNS species isolates showed 100% (95% CI: 95.6–99.6) resistance to Amoxicillin, Ampicillin, and Cefotetan antimicrobials. The result was relatively higher as compared to the report of Phophi et al. [46] who reported 90% and 89% of CoNS species resistance to ampicillin and penicillin antibiotics. In the current study, 54.2%,45/83( 95% CI: 42.9–65.2) of the CoNS species showed MDRS. The finding was relatively higher compared with the report of Phophi et al. [46], Mahato et al. [37] who reported 51% and 45.16% MDRS and lower than the report of Gizaw et al. [28] Central Oromia who reported 87.5% prevalence in CoNS species.

A large number (37.5%, 95% CI; 26.97–48.6) of coagulase-negative *Staphylococcus* species isolates were shown phenotypic methicillin resistance as compared to its genotypic (21.7%, CI: 13.4–32.1) methicillin resistance. The result was different from the report of Mahato et al. [37] in India reported 91.5% of CoNS isolates harboring the *mecA* genes; however, only 85.5% of the isolates were resistant to Oxacillin. The difference in prevalence of coagulase-negative *Staphylococcus* species developing multidrug resistance in the current study and other scholars’ reports might be associated with variations in disease epidemiology and the spread of drug-resistant CoNS species. Prolonged and inappropriate use of antimicrobials, creates selective pressure by depleting innocuous bacterial species and favors the flourishing of the pathogenic *Staphylococcus* species which in turn spread from one species to the other species through horizontal gene transfer [10, 57]. The difference in drug resistance capacity of the CoNS species isolates may also be attributed to variations in veterinary service delivery and farm hygiene which have a great contribution to the spread of drug-resistant CoNS species.

The CoNS species causes a wide range of human and animal diseases, and its pathogenicity is primarily due to a combination of toxin-mediated virulence, invasive capacity, and antibiotic resistance. The enterotoxin genes are responsible for the most potent staphylococcal food poisoning [11, 49].

The enterotoxin C (*sec*) gene was the only classical enterotoxin gene detected in CoNS species isolates in the present study. The finding was in agreement with the report of Homsombat et al. [33], Banaszkiewicz et al. [7], Nasaj et al. [40] who reported higher enterotoxin C (*sec*) gene prevalence in CoNS species-induced bovine mastitis. However, the result was different from the reports of Chajecka-Wierzchowska et al. [11], Helak et al. [32] who reported no classical enterotoxin genes detected from CoNS isolates.

The *hla* and *hlb* genes were detected in CoNS species at the rates of 8.4% and 9.6% respectively. The finding was much lower than the report of Pinheiro et al. [48] in Brazil, Nasaj et al. [41] in India reported 91.7% and 47.3%, 94.6% and 92.9% carriage of *hla* and *hlb* genes respectively. The discrepancy in the prevalence of the hemolysin genes might be associated with the difference in disease epidemiology [4], sample number, and sample source as well as the laboratory methods used for CoNS species virulent gene detection.

The *mecA* gene was the second most frequently detected (21.7%, 18/83) virulent gene in CoNS species isolates. The result infers a significant number of CoNS species harboring the methicillin resistance (*mecA*) gene, which poses a great health problem in animals and humans. The result was relatively in agreement with Seker et al. [51] in Turkey described 25% carriage of the *mecA* gene in CoNS species isolated from bovine milk. However, the finding was lower than the report of Xu et al. [60] in UK, Shrestha et al. [54] in Nepal, Ibadin et al. [34] in Nigeria, reported 29.5%, 70.7%, 30.5% carriage of *mecA* gene in CoNS isolates. A relatively lower report was made by Taponen et al. [56] in Finland from bovine mastitis milk, who reported 5% *mecA* gene carriage in CoNS species isolates.

The *pvl* gene was detected in 15/83, 18.07% of CoNS species isolates. The result was relatively higher than reports in India by Mahato et al. [37] who reported 6.5% *pvl* gene prevalence in CoNS species isolated from bovine milk and lower than Seker et al. [51] in Turkey reported 30.8% of CoNS species isolates were found carriage *pvl* gene.

Biofilm formation which is coded by the intracellular adhesion (*icaD*) gene is the major cause of drug resistance and persistent intramammary infections in bovine [52, 53]. Coagulase-negative *Staphylococcus* species were found to harbor 26.5% (22/83) of the *icaD* gene. The result was in agreement with the report of Gajewska and Chajecka-Wierzchowska [26] in central Poland showed carriage of 21.4% *icaD* genes. However, the result was lower than the report of Felipe et al. [21] on Argentinean dairy farms reporting 73.2% carriage of *icaD* in CoNS.

The difference in virulent gene profile in CoNS species between the present study and other reports might be associated with variations in the epidemiology of the study sites [4]. The knowledge gap on the rational use of drugs between veterinary practitioners might also play a great role in the spread of drug-resistant CoNS species [46]. Variations in CoNS species isolates virulent gene acquisition via horizontal gene transfer might also be another reason for the difference in the number of virulent genes detected from CoNS species isolates.

## Conclusions

Coagulase-negative *Staphylococcus* species were isolated from both apparently healthy and clinical mastitis-infected cow’s milk; which poses high risk to milk consumers and persons in contact with them. A large number of coagulase-negative *Staphylococcus* species isolates were found multidrug-resistant (54.2%) and carriage of methicillin resistance genes. Various virulence and food poisoning factors were detected from CoNS species isolates; but, the pathogenic impact of these virulence factors for intramammary infections is not well studied. Usage of antimicrobial like Gentamycin and Oxacillin and proper boiling of milk before consumption is advisable to reduce the impact of coagulase-negative *Staphylococcus* species. Performing virulent gene protein expression, case-control studies and whole-genome sequencing is the limitation of this study and should be studied in the future.

### Electronic supplementary material

Below is the link to the electronic supplementary material.

Supplementary Material 1

## Data Availability

The raw data generated during the study was attached along with this manuscript as a supporting file and further information on the data generated can be obtained upon the request of the corresponding author.

## Abbreviations

CLSI: Clinical Laboratory Standard Institute
CoNS: Coagulase-negative *Staphylococcus*
CSA: Central statistical agency
IMI: Intramammary infection
MDRS: Multidrug resistance *Staphylococcus*
MHA: Muller Hinton Agar
MSA: Mannitol Salt Agar
NMC: National Mastitis Council
