# Supplementary material for: Coagulase-negative staphylococci from bovine milk: Antibiogram profiles and virulent gene detection
Source: BMC Microbiol. 2024 Jul 18;24:263. doi: 10.1186/s12866-024-03415-0 (PMC11256419; doi:10.1186/s12866-024-03415-0)
Supplement: Supplementary file 1 — Supplementary Material 1 [file 12866_2024_3415_MOESM1_ESM.docx]

**Additional files 1. All Laboratory procedures employed to isolate and identify Coagulase-negative *Staphylococcus* species as well as the antimicrobial susceptibility test and the molecular detection of virulent genes from Coagulase-negative Staphylococcus species illustrated as follows.**

Annex1: Coagulase-negative *Staphylococcus* species Isolation Techniques

1.1. Isolation on Mannitol salt agar (MSA)

Mannitol salt agar (HiMedia Laboratories, Pvt. Ltd, India) was used for the selective growth of the *Staphylococcus* species. Mannitol salt agar has high salt concentrations (>7%), enhancing the selective growth of the *Staphylococcus* and *Micrococcus* species.

*Procedure*:

1. The MSA culture media was prepared based on the manufacturer’s instructions (111.02 grams of mannitol salt agar was diluted with 1 liter of distilled water).
2. The media was sterilized by the autoclave for 15 minutes under 121 pressures.
3. About 17 ml of mannitol salt agar was dispensed onto a sterile Petri-dish
4. The milk sample taken from the field was directly cultured on MSA and incubated for 24-48 hr at 37^o^C.
5. The shape, consistency, and color of the Staphylococcal colony and the color of the media were appreciated after 24hr as indicated in Figure 1.


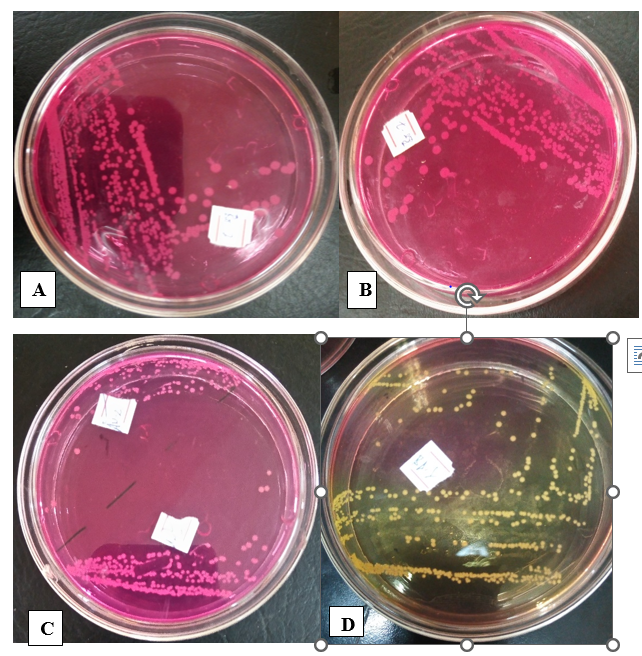
Figure 1: Characteristics of *Staphylococcus* species colonies grown on mannitol salt agar. Result: In terms of color, as it has seen in the figure above the *Staphylococcus* species grown on Mannitol salt agar has different characteristics, a white opaque colony (C), pink colony with pink medium ( A & B). Some CoNS species isolates showed yellowish color colonies with yellowish medium(D). Up on picking up of with inoculating loop, the *Staphylococcus* species showed different consistency; some *Staphylococcus* species grown on the mannitol salt agar had been observed with chalky consistency, some were slightly gelly, some had a very gelatinous consistency and some had mucoid consistency.

1.2. Coagulase-negative *Staphylococcus* Species Isolation and Identification on Blood Agar

The blood agar (HiMedia Laboratories, Pvt. Ltd, India) was prepared based on the manufacturer’s instructions.

1. By considering the media dissolving rate of 40 grams of blood agar with 1000 ml of distilled water) and sterilized with an autoclave for 15 minutes at 121 lb pressure).

2. After cooling the media below 50 ^o^C, 7% sheep blood was added

3. Upto 17ml of blood agar was dispensed to each sterile Petri dish and waited some time to cool.

4. A *Staphylococcal* species colony isolated on mannitol salt agar was subcultured onto the blood agar and incubating it at 37 ^o^C for 24-48 hours.

5. Variation in the hemolysis pattern between the *Staphylococcus* species observed as indicated in figure 2 below.


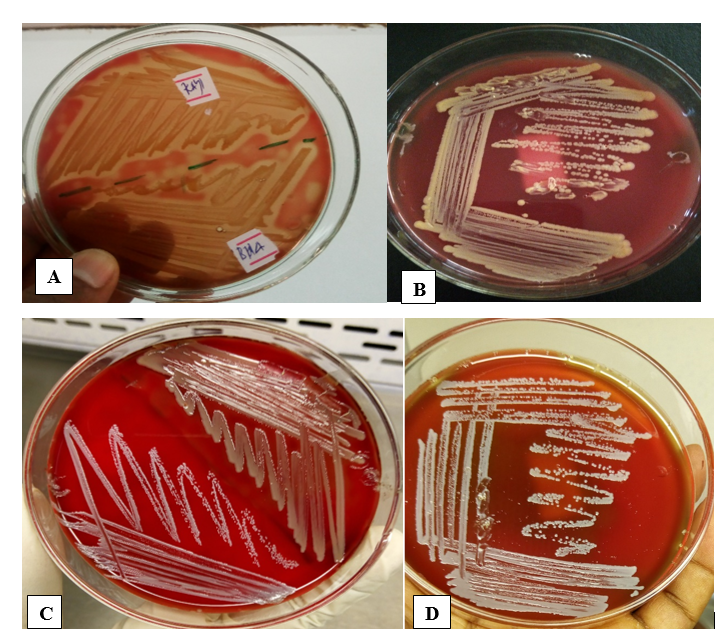


Figure 2. *Staphylococcus* species colonies, hemolysis pattern, and colony morphology difference on the blood agar. Some *Staphylococcal* species isolates show complete hemolysis on the blood agar (A); Some of them show a golden yellow colony color without any hemolysis pattern (B), Some of them show whitish colonies without any hemolysis pattern on the blood agar (C & D).

Annex 2: Coagulase negative *Staphylococcus* species Identification Techniques

The *Staphylococcus* species isolated by plate culturing on mannitol salt agar and blood agar were further differentiated by Gram staining, catalase test, OF test, coagulase test, sugar fermentation test, oxidase test, and Voges-Proskauer test.

2. 1. *Staphylococcus* species Identification using OF-medium

The OF medium was used to differentiate aerobic microbes from facultative anaerobes and anaerobic microbes. Thus, the bacteria that were grown on selective media mannitol salt agar were cultured on the OF medium to differentiate the *Staphylococcus* species from the *Micrococcus* species.

*Procedure*

1. 10 grams of lactose, sucrose, and dextrose sugars were measured and dissolved separately in 100 ml of distilled water and the sugar solutions were sterilized by a 0.45 µl pore size filter membrane.
2. The OF media was prepared separately in 3 beakers by mixing 2.4 grams of OF medium (HiMedia Pvt. Ltd., India) with 100 ml of distilled water and sterilized by the autoclave at 121 lb for 15 minutes.
3. After cooling the OF medium to 50 ^o^C 10 ml of the sugar solution was added separately to 100 ml of the OF medium.
4. The OF media containing sugar was mixed in a single beaker
5. The OF media was dispensed to the test tube
6. Each *Staphylococcus* species isolate was cultured in two test tubes containing the OF media, then after sealing one tube with paraffin oil and the other tube remain unsealed.
7. Incubate the tube at 37 ^o^C for 24-48 hours
8. Observe fermentation reaction in both tubes for each suspected *Staphylococcus* species isolates.
9. If fermentation takes place, on both paraffin oil sealed and unsealed test tubes, the isolate is considered as *Staphylococcus* species as shown in figure 3 B, but if fermentation takes place on the unsealed tubes only, the isolate was considered as *Micrococcus* species as shown in figure 3 A.


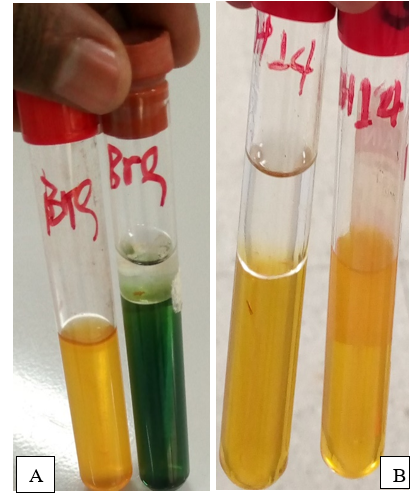


Figure 3. The OF test for the differentiation of *Staphylococcus* species from *Micrococcus* species. Figure 5 A fermentation reaction takes place only on paraffin oil unsealed tubes only, indicative of *Micrococcus* species whereas in figure 5 B in fermentation reaction takes place on both paraffin oil sealed and unsealed tubes indicating facultative anaerobic *Staphylococcus* species.

2.2. Gram Staining Techniques for *Staphylococcus* species Differentiation

Principle: The cell wall of Gram-positive bacteria is made up of a thick peptidoglycan layer, It firmly anchors the first dye, crystal violate, during the staining process and is not easily decolorized by acid alcohol, and it remains blue during counterstaining with Safranin or carbol-fuchsin dyes whereas Gram-negative bacteria’s have thin peptidoglycan layer, unable to firmly anchor the first staining dye and easily decolorized with acid alcohol, thus it becomes red during counter staining with Safranin or carbol-fuchsin dyes.

*The procedure of Gram Staining*

1. The bacterial smear was made on a clean grease free slide and allowed to dry

3. Crystal violet was poured and held for 30 seconds and rinsed with water

4. Gram's iodine was added and waited for 1 minute and rinse with water

5. Then 95% ethanol-alcohol was added and waited for about 20 seconds and rinsed with water.

6. Safranin was added as a counter stain and waited for 1 minute and rinsed with water.

7. Allowed to air dry, and microscopic inspection of the smear conducted under oil emulsion 100x objective.


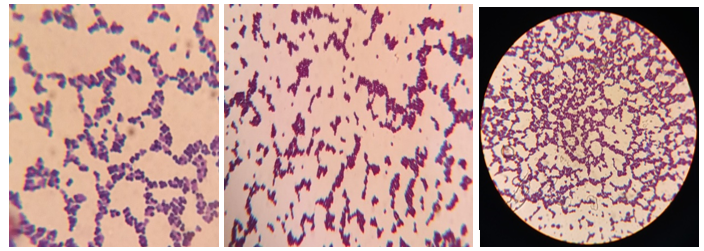


Figure 4. *Staphylococcus* species appearance during Gram staining techniques

2.3. Catalase test for *Staphylococcus* species Identification

***Principle***

The presence of catalase enzyme in bacteria catalyzes the release of oxygen from hydrogen peroxide (H_2_O_2_). It is used to differentiate those bacteria that produce catalase enzyme, such as staphylococci*,* from non-catalase-producing bacteria such as streptococci*.*

***Procedure***

1. Place a drop of 3% H_2_O_2_ on the glass slide
2. Using a sterile loop a small amount of colony grown on nutrient agar was mixed with hydrogen peroxide.
3. Oxygen bubbles were observed as a result of the breakdown of H_2_O_2_ to H_2_O and O_2_ As shown in figure 4


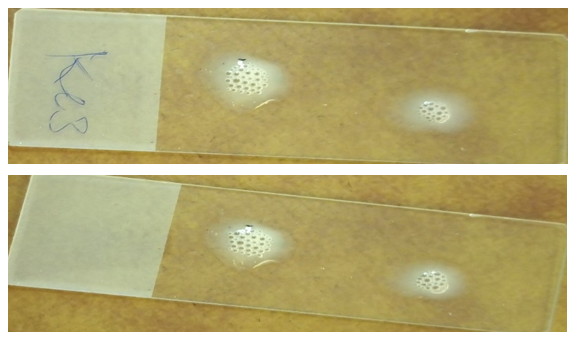


Figure 5. The *Staphylococcus* species isolate catalase test result

2.4. *Staphylococcus* species Identification using Coagulase Test

The Coagulase test was performed to differentiate coagulase-positive and coagulase-negative *Staphylococcus* species based on their ability to clot the rabbit plasma by converting plasma fibrinogen to fibrin. The test was performed based on the standards stated by Katz (2016). The slide coagulase test was conducted to detect the presence of bound coagulase enzyme-producing *Staphylococcus* species.) was used for the coagulase test.

2.4.1. Procedures of slide coagulase test

A slide coagulase test was used to detect bound coagulase enzymes produced by some Staphylococcus species like the *Staphylococcus aureus.*

1. The *Staphylococcus* species isolates were sub-cultured on trypticase soya agar (Hi Media Laboratories, India) for 18 hours

2. A drop of rabbit plasma (National veterinary institute (NVI), Debre Zeit, Ethiopia) was put on grease free microscopic slide

3. A drop of saline was put on the other side of the slide as a control

4. A pure bacterial colony was mixed up with a drop of rabbit plasma as a test and saline as a control

5. Observe the reaction of clotting within 10 minutes in contrast to the controls

6. Recording the positive result and proceed with the tube coagulase test for the negative result for the slide coagulase test.


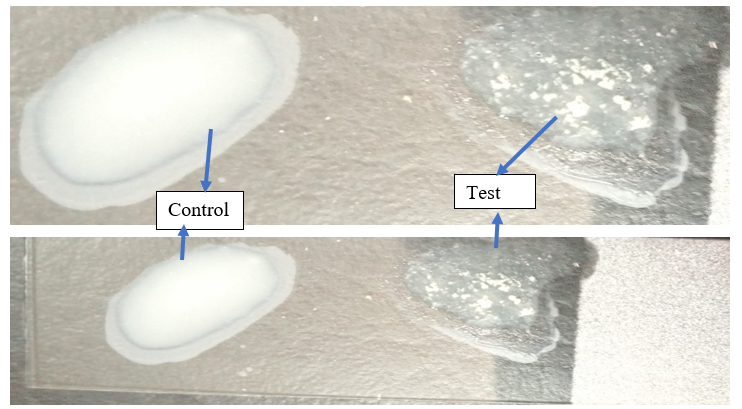


Figure 6. Slide coagulase test

2.4. 2. Tube coagulase test

The tube coagulase test is a more sensitive and accurate test for the differentiation of coagulase-positive and coagulase-negative *Staphylococcus* species. The test is used to detect the presence of free coagulase released from *Staphylococcus* species.

*The procedure of the tube coagulase test*

1. 0.5 ml of rabbit plasma was added to each test tube and labeled with the *Staphylococcus* species isolate code.

3. Then up to three Staphylococci isolated colonies were added from each isolate to the test tubes containing rabbit plasma

4. After mixing, incubate the test tubes at 35-37 ^o^C

5. The test was observed for clotting after 1 hour of incubation, then re-observed after 4 hours of incubation, and finally after 24 hours of incubation.

6. *Staphylococcus* species isolate that was clotting the rabbit plasma, considered as coagulase-positive *Staphylococcus* species as indicated in figure 6 A & B, and those Staphylococcal isolates which did not form a clot (C) after 24 hr considered as coagulase-negative Staphylococcus species as indicated in figure 6 ^o^C.


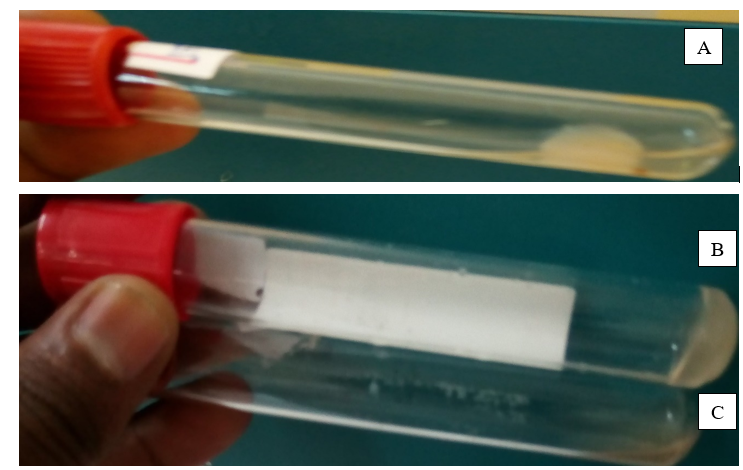


Figure 7. The tube coagulase test to differentiate coagulase-positive *Staphylococcus* species from coagulase-negative *Staphylococcus* species

2.5. Sugar fermentation test for Coagulase negative *Staphylococcus* species Differentiation

A sugar fermentation test was used to differentiate the *Staphylococcus* species based on their capacity of fermenting sugar and change the phenol indicator to yellow color, those *Staphylococcus* species which were not ferment sugar doesn’t change the phenol indicator and the media.

*Sugar fermentation test Procedure*

1. 1 gram of maltose and sucrose sugar was measured and added to a separate beaker containing 100 ml of nutrient broth (Guangdong Huanki microbial sci. and tech.co.Ltd) and labeled the beaker as sucrose and maltose
2. The media was sterilized with the autoclave under 121 lb for 15 minutes
3. Inoculate each test organism for both maltose and sucrose broth media
4. Incubate it for 24 hrs under 37 ^o^C
5. Phenol red reagent was added and color change was oserved as it has seen in figure 8 below.


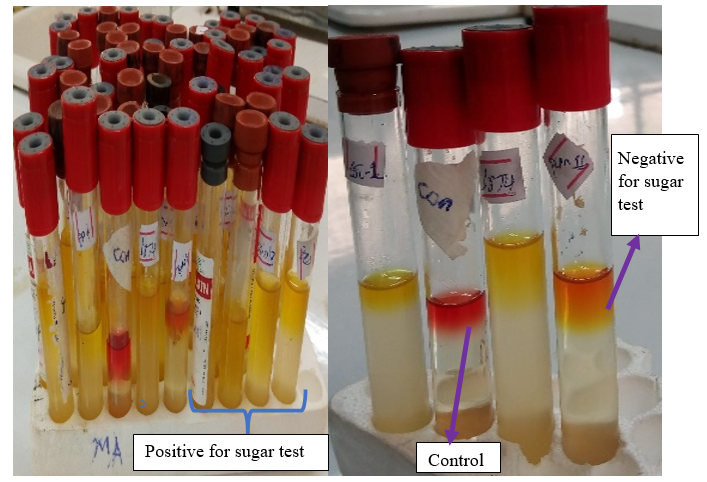


Figure 8. Maltose and sucrose tests for the differentiation of coagulase-negative *Staphylococcus* species. Result: The test that was changed to yellow after the addition of phenol red reagent, indicates the *Staphylococcus* species isolate was positive for the sugar fermentation test whereas if the test were changed to red after the addition of phenol red, it was considered as negative for the test as shown in figure 8 above.

2.6. Coagulase-negative *Staphylococcus* species differentiation using urease test

The urease test is used to differentiate coagulase-negative *Staphylococcus* species based on their ability to hydrolyze urea to ammonia and CO_2_ by using the urease enzyme. The formation of ammonia alkalizes the medium, and the p H shift is detected by the color change of phenol red from light orange at pH 6.8 to pink at pH 8.1.

The procedure of urease test.

1. The urea agar base (Accumix, Microxpress, India) was measured, by considering a 24-gram urea base dissolved in 950 ml of distilled water.
2. The urea agar base was sterilized by autoclave for 15 minutes under 121 lb
3. Filter membrane sterilized 50 ml of 40% urea solution was added to 950 ml of urea agar base solution after cooling it to 45^o^C.
4. The urea agar was dispensed to each test tube and the tube was put at a 45-degree inclination till solidified
5. The test organism was inoculated at the agar slant part and left butt un-inoculated for color change comparison.


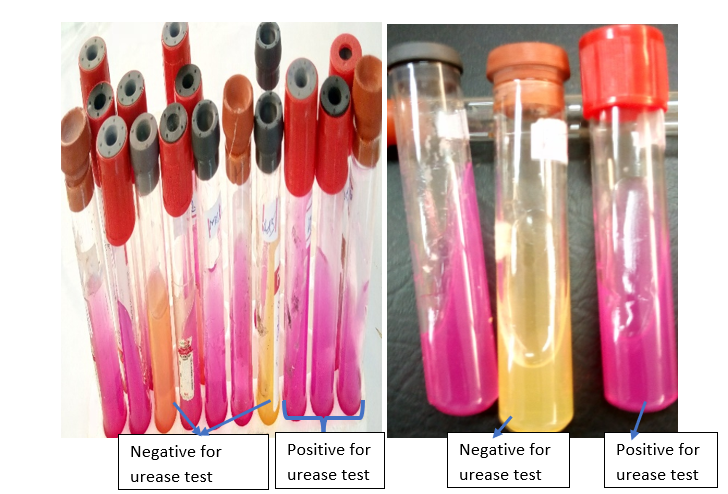


Figure 9. The urease test result of *Staphylococcus* species. Result: The *Staphylococcus* species that utilized the urea agar changes the media to pink color and if the *Staphylococcus* species didn’t hydrolyze urea agar the media remain yellow color as indicated in figure 9 above.

2.7. Oxidase test

The oxidase test detects the presence of a cytochrome oxidase system, which catalyzes electron transport between electron donors in bacteria and a redox dye called tetramethyl-p-phenylene-diamine. The dye has been reduced to a dark purple color.

*The procedure of the Oxidase test*

1. The Staphylococcus species were cultured in nutrient agar for 24 hours at 37^o^C.
2. A sterile cotton swab was immersed with an oxidase reagent
3. A pure colony of nutrient agar was taken using inoculating loops and struck over the cotton swab immersed with the oxidase reagent.
4. Observe the presence of deep blue color formed between the *Staphylococcus* species isolates

Result: the development of deep blue color on the cotton swab indicates oxidase-positive *Staphylococcus* species (Fig 10).


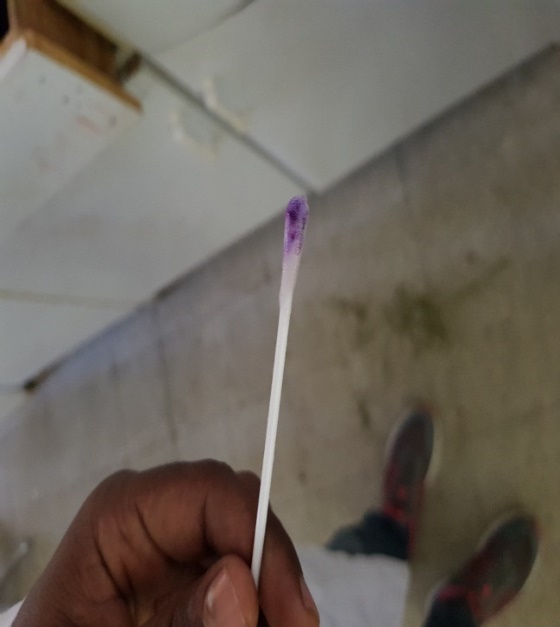


Figure 10. Oxidase test for differentiation of coagulase-negative *Staphylococcus* species isolates. Result: The development of deep blue color when the colony struck over the swab immersed with the oxidase reagent indicates, the *Staphylococcus* species were oxidase positive.

Annex 3. *Staphylococcus* species Virulent Gene Molecular Detection

3.1. DNA Extraction Protocol

*Procedure*

1. A pure colony of the Staphylococcus species was cultured for 24 hours in brain heart infusion broth (BHI) at 37^o^C for 24 hrs.
2. The bacterial pellet was prepared by transferring 1ml of Staphylococcal bacterial suspension grown in the BHI broth and centrifuged at 10,000 rpm for 10 minutes. The pellets were washed with 1ml of phosphate buffer saline solution by spinning at 10,000 rpm for 10 minutes and discarding the supernatant completely.
3. The bacterial genomic DNA extraction was performed using the EZ-10 spin column genomic DNA minipreps kit (Bio Basic Inc, Canada).
4. The prepared Staphylococcal bacterial pellet was re-suspended with 200µL of TE buffer and 400µL of digestion solution was added and well mixed; then 3µl of proteinase K was added and incubated at 55^o^C for 5 minutes.
5. After incubation 260 µl of 100% ethanol was added and well mixed; then after the mixture was applied to the EZ-10 spin column that is placed on 2 ml of collection tube and Spun at 10,000 rpm for 2 minutes.
6. The flowthrough in the collection tube was discarded and 500 µl of washing solution was added to the spin column and spun at 10,000 rpm for 2 minutes.
7. Again 500 µl of washing solution was added and spun at 10,000 rpm for 2 minutes.
8. Additional spinning was conducted for 2 minutes to remove the residuals of the washing solution.
9. The EZ-10 column was placed on a sterile 1.5 ml Eppendorf tube and 50 µl of elution buffer was added into the center of the membrane of the spin column and incubated at 50 ^o^C for 3 minutes.
10. To elute DNA from the column to the Eppendorf tube, the mixture was spun at 10,000 rpm for 2 minutes.
11. Then the extracted DNA was placed at -20 ^o^C until molecular work proceeded.
12. The quality of extracted DNA was assessed by a Nanodrop device at a 260 UV absorption rate and the quantity of DNA was also checked by gel electrophoresis.

3.2. Gel electrophoresis Procedure

1. 1.5% of the agarose gel (HiMedia Laboratories Pvt. Ltd., India) was measured and diluted with 1xTAE buffer

2. The mixture was boiled by the microwave until proper mixing assured

3. After cooling to 50^o^C, 10µl ethidium bromide was added to 200ml of agarose solution and mixed well then the agarose gel was dispensed on the gel try after placing the comb.

4. After solidifying the gel was transferred to the gel tank holding the TAE buffer

5. About 10 µl of the extracted DNA sample was mixed with 3µl loading buffer

6. The DNA-loading buffer mixture was transferred into the well of the gel

7. 5µl of DNA marker was added to one well

8. The anode of the gel try was connected to the anode of the power supply and the cathode of the gel try was connected to the cathode of the power supply and the current-voltage was adjusted to 150 V for 2 hours.

9. The movement of the DNA from the cathode (negative charge) to the anode (positive charge) was checked every 15 minutes.

10. After DNA moved about 3/4 of the gel, the gel was picked up from the gel try and the result of the amplified DNA was observed at the gel-doc system (UVITEC, Cambridge, UK).

11. Record the gel doc image using the software keyboard as shown in the figure below.


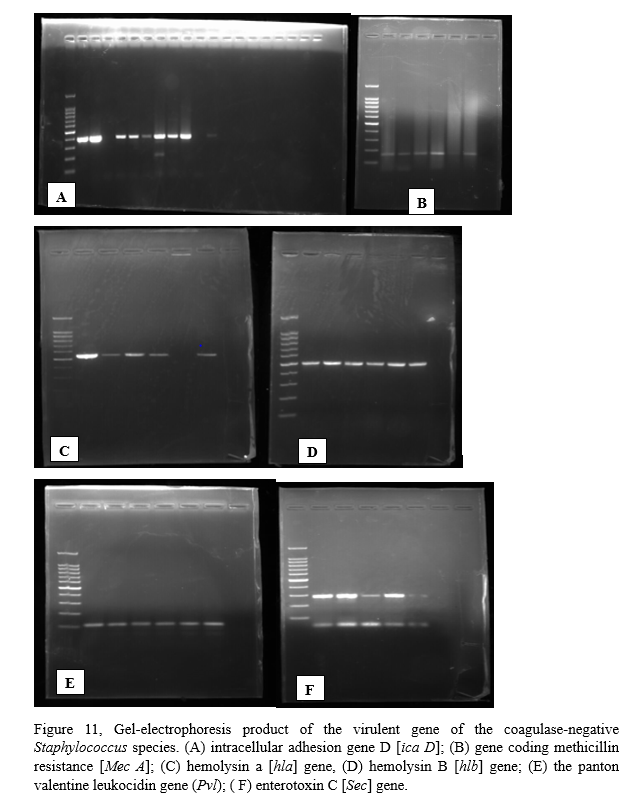


Annex 4. *Staphylococcus* species Antimicrobial Susceptibility Test

Antibiotic susceptibility testing is an in vitro test of the bacterial response to antibiotics. Its goal is to assist clinicians in selecting a specific antibiotic to treat a bacterial infection and to use the data to track bacterial resistance to antibiotics.

*Procedure*

1. Subculture pure coagulase-negative *Staphylococcal* species isolates in Nutrient agar (HiMedia pvt. Ltd, India) for 24 hours at 37^o^C.
2. Prepare the Mac Farland Standard
3. Mix 1.175 grams of barium chloride dihydrate (BaCl_2_) with 100ml of distilled water, and mix it gently
4. 1ml sulfuric acid (H_2_SO_4_). Was diluted with 99ml of distilled water
5. Measure 1.175% of 99.95ml of barium chloride solution and mix with 0. 05ml of 1% H_2_SO_4_.
6. Gently shaking and the turbidity used as 0.5 Mac-Farland standard
7. Preparation of the Muller-Hinton agar media (HiMedia pvt. Ltd., India)
8. Coagulase-negative *Staphylococcus* species inoculum preparation
9. 0.85% of sterile saline solution was added to a test tube coded with a specific *Staphylococcal* species isolates
10. Pure *Staphylococcal* species colony was taken from nutrient agar and mix it to the saline solution
11. The *Staphylococcal* species colony was continuously added to the saline solution until the turbidity was equivalent to 0.5 Mac-Farland standard.
12. A sterile cotton swab was immersed in turbid (0.5 Mac Farland standard equivalent) saline solution
13. Pick the swab by squeezing it against the wall of the test tube to remove excess fluid.
14. The *Staphylococcus* species isolates were cultured on MHA by rotating the swab over the surface of MHA and uniform culture was assured.
15. The MHA agar waited for 5 minutes till moisture is completely dried
16. The antimicrobial discs were placed on the MHA agar surface by keeping them 24 mm apart
17. Incubate it at 37 ^o^C for 24 hours
18. Measure the diameter of the zone of inhibition produced by each *Staphylococcus* species isolates using digital caliber and compared it with the CLSI standards
19. The result was interpreted as whether a typical *Staphylococcus* species isolate was susceptible, intermediate, or resistant to a particular antibiotic

Table 1. *Staphylococcus* species disc diffusion test cut of value result as of (CLSI, 2021) standards

| Antibiotic disc | *Staphylococcus* species | | | | | |
| --- | --- | --- | --- | --- | --- | --- |
|  | *S. aureus* | | *S. epidermidis* | | All Staphylococcus species | |
|  | S | R | S | R | S | R |
| Oxacillin (1 µg) | Not reliable | | ≥18 | ≤17 | - | - |
| Cefoxitin (30 µg) | ≥22 | ≤21 | ≥25 | ≤24 | ≥25 | ≤24 |
| Penicillin 10 units | ≥29 | ≤28 | ≥29 | ≤28 | ≥29 | ≤28 |
| Erythromycin(10µg) | ≥23 | ≤13 | ≥23 | ≤13 | ≥23 | ≤13 |
| Tetracycline (30µg) | ≥ 19 | ≤14 | ≥ 19 | ≤14 | ≥ 19 | ≤14 |
| Gentamycin (10 µg) | ≥15 | ≤12 | ≥15 | ≤12 | ≥15 | ≤12 |
| Rifampin (5 µg) | ≥ 20 | ≤16 | ≥ 20 | ≤16 | ≥ 20 | ≤16 |
| Ampicillin (AMP, 10, µg) | ≥29 | ≤28 | ≥29 | ≤28 | ≥29 | ≤28 |
| Vancomycin (VAN, 30 µg | Not reliable test for disc diffusion test | | | | | |

S- susceptible; R- resistance,


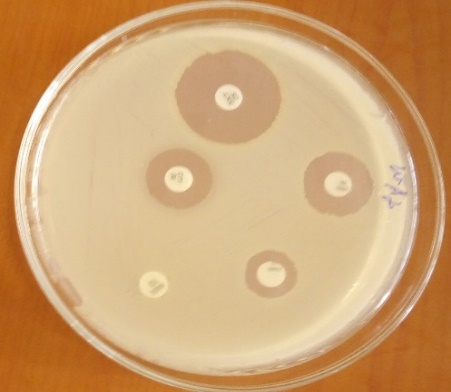

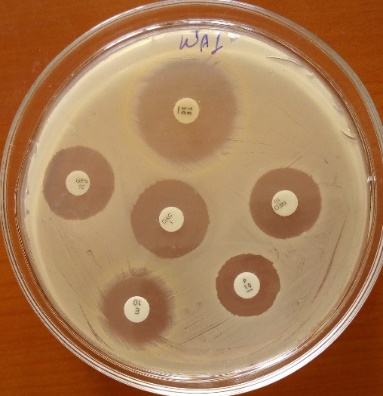

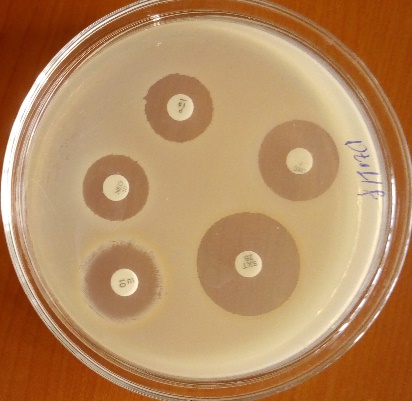


Figure 12. Antimicrobial susceptibility profiles of the coagulase-negative *Staphylococcus* species isolate
